# Why are leaves hydraulically vulnerable?

**DOI:** 10.1093/jxb/erw304

**Published:** 2016-09-06

**Authors:** Lawren Sack, Thomas N. Buckley, Christine Scoffoni

**Affiliations:** ^1^Department of Ecology and Evolutionary Biology, University of California Los Angeles, 621 Charles E. Young Drive South, Los Angeles, CA 90095, USA; ^2^IA Watson Grains Research Centre, Plant Breeding Institute, Faculty of Agriculture and Environment, The University of Sydney, Narrabri NSW 2390, Australia

**Keywords:** Drought, leaf extra-vascular conductance, leaf hydraulic conductance, leaf vein conductance, leaf vulnerability, shrinkage, vulnerability segmentation.

**As plant tissues dehydrate, water transport efficiency declines, a process typically attributed to air obstruction (embolism) in the xylem. Trifiló *et al*. (pages 5029–5039) dissect leaf hydraulic vulnerability and show that both xylem and living tissues may be important. If confirmed and clarified, an important role for outside-xylem hydraulic decline will change our understanding of how plants transport water and control biosphere carbon and water fluxes.**

The study of leaf hydraulics has taken off exponentially in the past decade, after many decades of very sparse attention, especially compared with stem hydraulics. The obstacle was that leaves seemed impossibly complex. Whereas in stems, water flows through the xylem, in leaves water flows not only through the xylem within the complex venation network but also across living tissues outside the xylem, and evaporates somewhere in the leaf, before diffusing through the stomata. No part of that system was well understood.

The first step was to establish methods to reliably quantify the hydraulic conductance of the whole leaf (*K*_leaf_), defined as the flow rate divided by a given gradient in water potential (Ψ) from petiole to mesophyll (reviewed by Sack and Tyree, 2005). Subsequent experiments on leaves with severed veins allowed the dissection of leaf xylem hydraulic conductance (*K*_x_) from outside-xylem conductance (*K*_ox_) and showed that the xylem and outside-xylem compartments contribute strongly and similarly to total hydraulic resistance on average across species, with the relative contributions highly variable among species (reviewed by Sack and Holbrook, 2006).

Now each component of the leaf hydraulic system could be illuminated by creative and exciting research, with major breakthroughs on the role of the venation network (reviewed by Sack and Scoffoni, 2013), the properties of the living tissues, including aquaporins, which affect leaf water transport (Maurel *et al.*, 2015), and the vapor phase transport pathways (Rockwell *et al.*, 2014; Buckley, 2015). These studies further showed that *K*_leaf_ is responsive to internal and external factors that affect both xylem and living tissues, such as temperature, light and water status. In this issue, Trifiló *et al.* (2016) extend the dissection approach to clarify the response of *K*_leaf_ to dehydration.

## Steep leaf response

The decline of *K*_leaf_ with dehydration is known as ‘vulnerability’, by analogy with concepts established for stem hydraulics. Because water flows through the xylem under tension, it is susceptible to interruption by air, which can fill the xylem conduit and reduce the volume for water flow. The decline in stem conductivity with dehydration due to embolism often only occurs in very dehydrated stems. Debates surrounding the quantification of stem xylem embolism, its frequency during diurnal transpiration and drought, and its impact on hydraulic conductance are a testament to the importance of the phenomenon across scales in biology, as stem xylem embolism is now recognized as a primary determinant of species’ maximum heights, drought tolerances and ecological distributions.

Meanwhile, tens of studies in the past decade have shown steep hydraulic declines in leaves with dehydration before wilting or turgor loss, using multiple measurement approaches applied to different species (Box 1). This response has typically been attributed, albeit with little direct evidence, to embolism in the xylem. A widespread decline of *K*_leaf_ under moderate dehydration would have major implications. Strong *K*_leaf_ vulnerability relative to stems would drive stomatal responses under soil and atmospheric drought and thereby control carbon and water fluxes from leaves and canopies, with feedback on the climate system (Sperry *et al.*, 2016).

**Box 1. Vulnerability of leaf hydraulic conductance (*K*_leaf_) to dehydration, showing strong declines before turgor loss point**

*K*_leaf_ vulnerability curves are shown for a sampling of nine diverse species (grey lines) ranging in drought tolerance. These were obtained as follows: *Hakea lissosperma* (HaLi), *Lomatia tinctoria* (LoTi) and *Tasmania lanceolata* (TaLa) from Blackman *et al.* (2010) – rehydration kinetics method; *Comarostaphylis diversifolia* (CoDi), *Heteromeles arbutifolia* (HeAr) and *Lantana camara* (LaCa) from Scoffoni *et al.* (2012) – evaporative flux method; and *Magnolia grandiflora* (MaGr), *Quercus rubra* (QuRu) and *Vitis labrusca* (ViLa) from Trifiló *et al.* (2016) – vacuum pump method. Turgor loss points are shown as black dots for each species. The average *K*_leaf_ vulnerability curve is shown (black line) with the average turgor loss point (grey dot).

**Figure:**
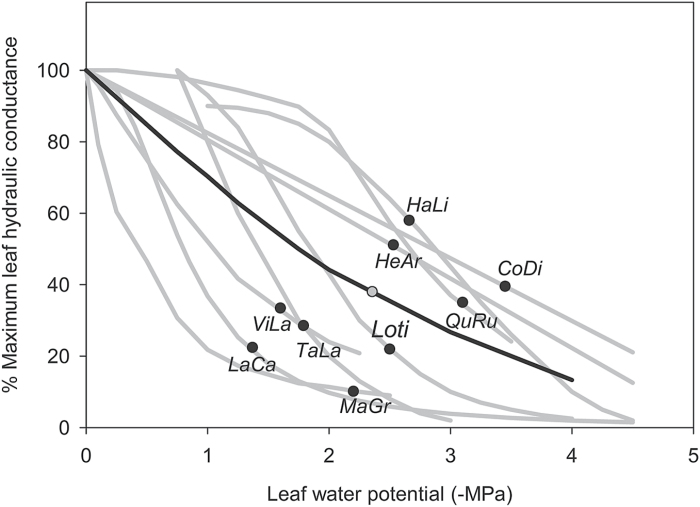

It is now imperative to determine what causes this steep leaf response, which can no longer be attributed *a priori* to xylem embolism. Trifiló *et al*. have applied rigorous refinements of previous methods to this problem. Importantly, they confirm the dramatic decline of *K*_leaf_ before turgor loss point and, following Hernandez-Santana *et al.* (2016), demonstrate similar vulnerability using two different methods on the same species. They also used a recently developed vacuum method based on measuring leaves with severed minor veins to measure the leaf xylem hydraulic vulnerability (Scoffoni and Sack, 2015), and determined the outside-xylem hydraulic vulnerability by subtraction. They found strong hydraulic decline in both xylem and outside-xylem compartments, with both contributing to *K*_leaf_ decline across four diverse species. They confirm that *K*_ox_ vulnerability is associated with tissue shrinkage outside the xylem (Scoffoni *et al.*, 2014), and speculate that this could protect the rest of the plant from embolism by driving a pre-emptive stomatal closure response to moderate dehydration.

## Xylem and outside-xylem pathways

This research is especially significant because it clearly shows the potential roles of both xylem and outside-xylem pathways in controlling *K*_leaf_ vulnerability and, by extension, whole-plant hydraulic conductance and productivity. These findings will change the way plant physiologists and ecologists think about water transport, with a major potential role for living tissues as well as xylem in control of the system.

This work raises the urgent need for additional work to confirm and extend the partitioning of *K*_leaf_ vulnerability. Trifiló *et al*. showed that in two species, *Aleurites moluccana* and *Vitis labrusca*, *K*_x_ and *K*_ox_ both played important roles in *K*_leaf_ decline, whereas in another two, *Magnolia grandiflora* and *Quercus rubra*, only *K*_ox_ played a role. These findings, based on measurements of the rehydration of previously dehydrated leaves and of vacuum-driven water uptake under low irradiance, need confirmation on leaves transpiring under high irradiance as would be typical during photosynthesis. *K*_leaf_ vulnerability depends on light, as water flow through living tissues is influenced by aquaporins that are responsive to both light and turgor (Maurel *et al.*, 2015), and as tissues absorb light, vapor phase transport may drive a great deal of water movement from the mesophyll to the transpiring epidermis (Guyot *et al.*, 2012; Rockwell *et al.*, 2014; Buckley, 2015). Thus, as Trifiló *et al*. recognized, the role of *K*_ox_ might be yet stronger under high irradiance.

Even more essentially, the method for determining *K*_x_ decline and the inference that embolism is not the key driver of *K*_leaf_ decline need to be validated, ideally with a visual method. A recently developed optical method (Brodribb *et al.*, 2016) similarly showed that xylem embolism begins late in dehydration, but given that method’s low resolution at fine scales and its inability to scan within the entire tissue cross-section, it might not show all the emboli that occur in all the veins. To prove the conclusion that *K*_ox_ decline can be the major driver of *K*_leaf_ vulnerability, it will be necessary to apply microCT (X-ray microtomography) to determine potential embolism in veins for species that have also been analyzed for *K*_leaf_, *K*_x_ and *K*_ox_ decline.

The causes of *K*_ox_ decline also need to be investigated. Given the complexity of *K*_ox_, these may relate to cell geometry changes during shrinkage, aquaporin activity and/or changes in the vapor phase transport. The application of models calibrated with anatomy will be an essential approach to determining the drivers of *K*_ox_ decline. Finally, speculation that *K*_ox_ decline may protect the xylem in the leaf and throughout the rest of the plant from tensions that induce embolism needs to be validated using models and measurements of whole-plant transport.

## Shift in understanding

If *K*_ox_ decline turns out to be the major driver of *K*_leaf_ decline, this will shift our understanding of water transport in the whole plant, and thus in the entire soil–plant–atmosphere continuum. No longer will water flow through soil or dead xylem cells be considered the major control points – rather, the living tissues outside the xylem, including the stomata, leaf vein parenchyma and leaf mesophyll, may take a central position. The light and turgor responses of these cells within leaves would then be recognized as influencing water transport and stomatal sensitivity, drought tolerance and productivity at plant and landscape scale. Outside-xylem hydraulic conductance will need to be explicitly incorporated into new models of plant water use, ecohydrology, landscape and global fluxes, and climate. The dissection of leaf hydraulic pathways would thus shift our understanding of how living plant cells and tissues influence the biosphere.
